# Safety review of hydroxyprogesterone caproate in women with a history of spontaneous preterm birth

**DOI:** 10.1038/s41372-020-00849-y

**Published:** 2020-10-14

**Authors:** Baha Sibai, George R. Saade, Anita F. Das, Jennifer Gudeman

**Affiliations:** 1 Department of Obstetrics, Gynecology, and Reproductive Sciences, McGovern Medical School-UTHealth, 6431 Fannin, Houston, TX 77030 USA; 2 Department of Obstetrics and Gynecology, University of Texas Medical Branch, University of Texas, Galveston, TX USA; 3 Das Consulting, Guerneville, CA USA; 4 AMAG Pharmaceuticals, Inc., Boston, MA USA

**Keywords:** Adverse effects, Outcomes research

## Abstract

17-alpha-hydroxyprogesterone caproate (17P) has been in use for prevention of recurrent preterm birth since 2003 when the Meis trial was published. A requirement for Food and Drug Administration approval of 17P was a confirmatory trial, called “PROLONG”, which was recently completed, but did not replicate the efficacy demonstrated in the Meis trial. This review analyzes the safety data from each trial, as well as integrated data from the two trials. The relative risks (95% CI) with 17P versus placebo in the integrated dataset were 0.66 (0.25–1.78) for miscarriage, 1.83 (0.68–4.91) for stillbirth, and 0.86 (0.53–1.41) for all fetal and neonatal death. The rate of gestational diabetes in the integrated dataset was 3.6% for 17P vs. 3.8% for placebo. Similar findings with low and comparable rates between 17P and placebo were also found for other adverse events. The integrated safety data demonstrate a favorable safety profile that was comparable to placebo.

## Introduction

Preterm birth (PTB) is recognized as the leading cause of neonatal morbidity and mortality in the United States (US) [1]. The US is ranked among the top ten countries in the world in total number of PTBs with a rate of PTB that is also considerably higher than other developed countries [2, 3].

Women who have had a prior spontaneous PTB (SPTB) have a 2.5-fold greater risk for subsequent SPTB than women with no prior history of PTB [4, 5]. A synthetic derivative of progesterone, 17-α-hydroxyprogesterone caproate (17P), has a history of use in pregnant women dating back approximately six decades [6]. Five small studies conducted between 1964 and 1985 were included in a meta-analysis in 1990, which found that 17P reduced the rate of PTB by 42% [7].

The National Institute of Child Health and Human Development Maternal Fetal Medicine Units (NICHD MFMU) network conducted a multi-center, double-blind, placebo-controlled trial evaluating 17P in women with a documented history of SPTB [8]. The trial, which was published in 2003 in the *New England Journal of Medicine*, was stopped early because of clear benefit of 17P [8]. The trial, hereafter referred to as the “Meis trial”, demonstrated that 17P significantly reduced the rate of recurrent PTB < 37, <35, and <32 weeks, and significantly prolonged the duration of pregnancy. For the primary outcome of PTB < 37 weeks, the rate was 36.3% in the 17P group compared to 54.9% in the placebo. Following publication of the Meis trial, the American College of Obstetricians and Gynecologists (ACOG) released guidelines recognizing the trial and noted that 17P was specially formulated for research and was not available commercially [9].

In 2011, FDA granted approval of Makena® (17P) to reduce the risk of recurrent PTB in women with a prior spontaneous singleton preterm birth. As a condition of FDA approval, a confirmatory trial, “Progestin’s Role in Optimizing Neonatal Gestation (PROLONG)” and a PROLONG infant follow-up study were required. The PROLONG trial has been completed while the infant trial is still on-going. There was relatively low (23%) enrollment in the US because 17P was considered standard of care in the US; as such, investigators were reluctant to randomize patients to a placebo-controlled trial. Thus, the PROLONG trial population was predominantly (77%) outside of the US, in countries with markedly lower background rates of PTB [10]. In contrast to the Meis trial, the PROLONG trial did not demonstrate a significant reduction in PTB with 17P; the incidence of PTB at <35 weeks was 11.0% in the 17P group vs 11.5% in the placebo groups. The sponsor has submitted a proposed Makena labeling update to FDA to include safety and efficacy from PROLONG. Following the publication of PROLONG, ACOG and the Society for Maternal Fetal Medicine (SMFM) released guidelines on October 25, 2019 [11, 12], where the medical societies recognized the different populations enrolled in the Meis and PROLONG trials and continue to support 17P utilization, using shared-decision making with patients. An FDA Advisory Committee was held on October 29, 2019 to discuss the totality of the available 17P data and consider whether 17P had “substantial evidence of effectiveness” [13]. In addition to efficacy, product safety also remains a cornerstone consideration. Thus, a thorough review and evaluation of the safety data from both the Meis and PROLONG trials, as well as their combined data (herein referred to as “Integrated Meis/PROLONG”), was undertaken to further inform the risk/benefit profile of 17P in women with a prior SPTB.

## NICHD MFMU Meis, PROLONG, and integrated trial safety data

### Trial designs

#### Meis

The Meis trial was a US multi-center, randomized, double-blind, placebo-controlled trial of 17P conducted by the NICHD MFMU [8], with enrollment between 1999 through 2002. Subjects with a documented history of SPTB were randomized to 250 mg of 17P (*N* = 310) or placebo (*N* = 153) by weekly IM injection beginning at 16–20 weeks/6 days of gestation and continuing until 37 weeks of gestation or delivery, whichever occurred first. The primary endpoint was reduction of PTB < 37 weeks of gestation. The Meis trial estimated that 500 subjects had an 80% power to detect a 33% reduction in the rate of preterm delivery (from 37% to 25%). The Meis trial was halted at the second interim analysis, based upon reaching the pre-specified criterion threshold of alpha = 0.015 for the primary outcome.

#### PROLONG

PROLONG was an international, multi-center, randomized, double-blind, placebo-controlled trial conducted by a pharmaceutical sponsor, with enrollment between 2009 and 2018 [10]. PROLONG was designed in conjunction with the FDA as a required confirmatory trial. Subjects with a documented history of SPTB were randomized to 250 mg of 17P (*N* = 1130) or placebo (*N* = 578) by a weekly IM injection beginning between 16 and 20 weeks/6 days of gestation and continuing until 37 weeks of gestation or delivery, whichever occurred first. PROLONG had two co-primary efficacy endpoints and was powered to detect a 30% difference for PTB <35 weeks gestation and a 35% difference in a neonatal composite index. Due to the numerically higher rate of miscarriage and stillbirth observed in patients treated with 17P compared to vehicle in the Meis trial, the PROLONG trial included a primary safety objective designed to rule out a doubling in the risk of fetal or early infant death in the 17P group compared to placebo. A “doubling of risk” was selected and agreed upon with FDA based on sample size consideration as well as clinical relevance given the expected low rate of the outcome. Assuming 4% fetal or early infant death rate in both treatment groups, a sample size of 1707 provided 83% power to rule out doubling in risk of fetal or early infant death. For context, to rule out a 1.5-fold increase in risk, the sample size needed would be ~4300.

### Safety: definitions and differences in collection between Meis and PROLONG

For the Meis trial, side effects related to study drug administration noted by the study coordinator (but not mentioned by the subject) were recorded as adverse events (AEs) on the Case Report Form. In addition, subjects were asked if they had any symptoms or side effects that the subject believed was associated with the study medication or medication administration (open ended question), as well as questions regarding specific AEs that were of interest to the investigators (i.e., “did you feel pain at the injection site?”). All deaths (maternal, fetal, or neonatal), life threatening events, and events that were serious and unexpected in nature, severity or frequency were also reported. Investigators did not assess non-serious AEs for severity or relationship to study drug. AEs were collected until the time of delivery and were coded using the Medical Dictionary for Regulatory Activities (MedDRA) Version 8.0.

For the PROLONG study, AEs were collected according to standard International Council for Harmonization (ICH) Good Clinical Practice guidelines. Each subject was asked for any medically related changes in their well-being. AEs were included if reported by the woman or observed by the investigator, with the investigator providing their assessment of causality (definite, probable, possible, unlikely/remote, or definitely not) and intensity (mild, moderate, severe) for all AEs. For PROLONG, AEs were recorded through 35 ± 7 days after the last dose of study drug and thus, some AEs occurring after delivery were recorded. AEs were coded using MedDRA Version 14.1.

### Adverse events

#### Meis trial

The AE profiles were comparable between the two treatment groups. The overall incidence of AEs was 57.7% for the 17P and 56.2% for the placebo groups. The most common type of AE reported was injection site events (pain, swelling, itching, and nodule formation) which occurred in 42.3% of patients randomized to 17P and in 38.6% of those randomized to placebo [14].

The AEs occurring in ≥2% of the 17P-treated patients, as well as those with a numerically higher rate than placebo are listed in Table 1 [14].

**Table 1:** Relevant obstetrical outcomes and events occurring in ≥2% of the women in the 17P group or at a higher rate in the 17P versus Placebo group.

	Meis ^a^		PROLONG ^b^		Integrated	
	17P *N* = 310 *n* (%)	Placebo *N* = 153 *n* (%)	17P *N* = 1128 *n* (%)	Placebo *N* = 578 *n* (%)	17P *N* = 1438 %	Placebo *N* = 731 %
Admission for preterm labor (other than delivery admission)	49 (16.0)^c^	21 (13.8)	187 (16.5)^c^	84 (14.5)	16.4^c^	14.4
Preeclampsia or gestational hypertension	27 (8.8)^c^	7 (4.6)	47 (4.2)^c^	30 (5.2)	5.2^c^	5.1
Nausea	18 (5.8)	7 (4.6)	55 (4.9)	26 (4.5)	5.1	4.5
Gestational diabetes	17 (5.6)^c^	7 (4.6)	35 (3.1)^c^	21 (3.6)	3.6^c^	3.8
Headache	4 (1.3)	0	68 (6.0)	28 (4.8)	5.0	3.8
Injection site pruritus	18 (5.8)	5 (3.3)	42 (3.7)	23 (4.0)	4.2	3.8
Injection site swelling	53 (17.1)	12 (7.8)	5 (0.4)	2 (0.3)	4.0	1.9
Back pain	4 (1.3)	1 (0.7)	50 (4.4)	20 (3.5)	3.8	2.9
Vomiting	10 (3.2)	5 (3.3)	42 (3.7)	19 (3.3)	3.6	3.3
Urticaria	38 (12.3)	17 (11.1)	5 (0.4)	0	3.0	2.3
Constipation	2 (0.6)	1 (0.7)	38 (3.4)	17 (2.9)	2.8	2.5
Insomnia	2 (0.6)	1 (0.7)	36 (3.2)	13 (2.2)	2.6	1.9
Cervical incompetence/cerclage	5 (1.6)	2 (1.3)	34 (3.0)	16 (2.8)	2.4	2.2
Injection site nodule	14 (4.5)	3 (2.0)	18 (1.6)	9 (1.6)	2.2	1.6
Diarrhea	7 (2.3)	1 (0.7)	23 (2.0)	13 (2.2)	2.1	1.9
Oligohydramnios^d^	11 (3.6)^c^	2 (1.3)	9 (0.8)^c^	12 (2.1)	1.4^c^	1.9
Chorioamnionitis^d^	11 (3.6)^c^	5 (3.3)	9 (0.8)^c^	2 (0.3)	1.4^c^	1.0
Cholestasis^d^	0	0	3 (0.3)	5 (0.9)	0.2	0.7
Maternal depression^e^	0	0	5 (0.4)	5 (0.9)	0.3	0.7
VTE^d^	1 (0.3)	0	0	1 (0.2)	0.07	0.1

*17P* 17-α-hydroxyprogesterone caproate, *VTE* venous thromboembolism.

^a^References [8, 25, 26].

^b^Reference [10].

^c^*N* = 306 as denominator for these AEs for Meis; *N* = 1130 as denominator for these AEs for PROLONG.

^d^Included in table given medical relevance for this therapeutic class.

^e^Included in table given reference in the product insert. Maternal depression was based on AE reporting.

The severity and the causality of non-serious AEs were not evaluated in the Meis trial. A low percentage of subjects discontinued therapy due to potential AEs (2.2% vs 2.6% in the 17P vs placebo groups, respectively). The most common adverse events that led to the discontinuation in either group were urticaria and injection site pain/swelling.

#### PROLONG

The AE profiles between the two treatment groups were comparable. There were 57.3% and 57.8% of subjects with at least one AE in the 17P and placebo group, respectively. Most AEs were mild, and most were considered unrelated to study drug by the investigator at the research site. The most frequently reported AEs in either group were anemia (9.2% in 17P and 9.7% in placebo) and headache (6.0% in 17P and 4.8% in placebo) [15].

Treatment-related AEs were reported for 10.8% and 9.2% of 17P and placebo subjects, respectively. The only treatment-related AEs reported by ≥2.0% of subjects in either treatment group were injection site pain (2.8% in 17P vs 3.6% in placebo) and injection site pruritus (3.5% in 17P vs. 3.6% in placebo).

There was a low percentage of AEs leading to study drug withdrawal; 11 subjects (1.0%) in the 17P vs 5 (0.9%) in the placebo group. Four of the eleven 17P subjects withdrew due to injection site events; two of the five placebo subjects group withdrew due to cholestasis [16].

### Integrated Meis/PROLONG

Table 1 lists the relevant obstetrical outcomes and common adverse events (≥2% of subjects in the 17P group or at a higher rate in the 17P group). Based upon the pharmacology of 17P and/or FDA-required labeling for progestogens, the AEs referred to as venous thromboembolism and cholestasis were also considered clinically relevant and included in Table 1. When the data from both the Meis and PROLONG trial were integrated, adverse events occurred at low rates that were comparable between the 17P and placebo groups. A total of 1.3% of subjects receiving 17P were reported as discontinuing therapy due to AEs compared to 1.2% of those receiving placebo. The most common AEs that led to discontinuation in both groups were urticaria and injection site pain/swelling (1% each).

### Serious adverse events

#### Meis trial

There were four serious adverse events (SAE) in this trial, all in the 17P group: pulmonary embolus, injection site cellulitis, an infant with possible hypogonadism and a subject who had a stillbirth and developed postpartum hemorrhage and respiratory distress after delivery.

#### PROLONG

Overall, 34 (3.0%) subjects in the 17P group and 18 (3.1%) in the placebo group experienced SAEs. The most frequently reported SAEs in the 17P group were premature separation of the placenta (0.4%), placental insufficiency (0.4%), and pneumonia (0.3%). In addition, *Escherichia coli* sepsis, pyelonephritis, and wound infection were each reported in two subjects in the 17P group. The most frequently reported SAE for subjects in the placebo group were cholestasis (0.5%), and premature separation of the placenta (0.3%) [15].

Two subjects had an SAE considered possibly related to study treatment (one subject in the 17P group had nephrolithiasis considered possibly related and one subject in the placebo group had severe cholestasis considered probably related) [15].

### Integrated Meis/PROLONG

Given the relative infrequency of SAEs from both the Meis and PROLONG trials, an integrated analysis of the SAEs is not presented.

### Maternal adverse events of interest

#### Gestational diabetes mellitus (GDM)

There was a numerically higher rate of GDM in the 17P group compared to placebo in the Meis trial. However, the reverse pattern was reported in PROLONG, where the rate of GDM was numerically higher in the placebo group. As the US has higher background rates of GDM compared to other countries enrolling women in PROLONG, a US PROLONG only subgroup was also assessed, and the GDM rates were comparable between the 17P and placebo groups (7.4% in 17P vs. 7.5% in placebo). Integrated data of the two trials indicated comparable rates between 17P and placebo (Table 1).

#### Hypertension/preeclampsia

There was a numerically higher rate of HTN/preeclampsia reported in the 17P group than placebo in the Meis trial. However, in PROLONG, the reverse pattern was reported with the rate of HTN/preeclampsia numerically higher in the placebo group. Integrated data of the two trials indicated comparable rates between the two groups (Table 1).

#### Venous thromboembolism (VTE)

There were two cases of VTE reported in the integrated dataset: one case in the 17P group in the Meis trial and one case in the placebo group from PROLONG (Table 1).

#### Cholestasis

No cases of cholestasis were reported in the Meis trial. In PROLONG, a limited number of cholestasis was reported in both the 17P and placebo groups (Table 1).

### Perinatal deaths

A miscarriage was defined as fetal loss <20 weeks gestation in both trials. Since subjects could be enrolled in either trial up to 20 weeks/6 days, only subjects randomized prior to 20 weeks were considered eligible to have a miscarriage. A stillbirth was defined as loss of the pregnancy after 20 weeks of gestation. Neonatal death was defined as death within 28 days after delivery. As requested by FDA for PROLONG, “early infant death” was a category specifically assessed, defined as death among liveborn neonates born between 20 and 24 weeks of gestation; this was a subset of the neonatal death category.

#### Meis trial

Table 2 provides the reported rates for miscarriage, stillbirth, and neonatal death from the Meis trial. The investigators at the research sites classified all of these as not related to treatment.

**Table 2:** Fetal and neonatal death.

	Meis^a^		PROLONG^b^		Integrated	
	17P *n*/*N* (%)	Placebo *n*/*N* (%)	17P *n*/*N* (%)	Placebo *n*/*N* (%)	17P *n*/*N* (%)	Placebo *n*/*N* (%)
Miscarriage^c^	5/209 (2.4)	0/107 (0)	4/866 (0.5)	7/448 (1.6)	9/1075 (0.8)	7/555 (1.3)
RR (95% CI)	NC		0.28 (0.08–0.94)		0.66 (0.25–1.78)	
Stillbirth ^d^	6/301 (2.0)	2/153 (1.3)	12/1124 (1.1)	3/571 (0.5)	18/1425 (1.3)	5/724 (0.7)
RR (95% CI)	1.56 (0.31–7.80)		2.07 (0.59–7.29)		1.83 (0.68–4.91)	
Neonatal death^e^	8/295 (2.7)	9/151 (6.0)	6/1093 (0.5)	3/559 (0.5)	14/1388 (1.0)	12/710 (1.7)
RR (95% CI)	0.44 (0.17–1.13)		0.98 (0.24–3.91)		0.60 (0.28–1.28)	
Fetal/neonatal death^f^	19/310 (6.1)	11/153 (7.2)	22/1128 (2.0)	13/578 (2.3)	41/1438 (2.9)	24/731 (3.3)
RR (95% CI)	0.85 (0.42–1.75)		0.85 (0.43–1.67)		0.86 (0.53–1.41)	

Relative risk is unadjusted for Meis, adjusted for gestational age at randomization stratum in PROLONG and adjusted for study in the integrated analysis.

*NA* not applicable, *NC* not calculable, *RR* relative risk, *17P* 17-α-hydroxyprogesterone caproate, *CI* confidence interval.

^a^References [25, 26].

^b^Reference [10].

^c^Denominator is number of subjects who received study drug and were randomized 20^0/7^weeks of GA. Miscarriage was defined as spontaneous delivery from 16^0/7^ to 19^6/7^ weeks of gestation.

^d^Denominator is number of subjects who received study drug and were pregnant beyond ≥20^0/7^ weeks of GA. Stillbirth was defined as antepartum or intrapartum death from 20^0/7^ weeks of gestation through term.

^e^Denominator is number of subjects who received study drug and did not have a miscarriage or stillbirth. Patients with missing data are assumed not to have the specified outcome.

^f^Denominator is number of patients who received study drug.

#### PROLONG

As requested by the FDA, PROLONG had a primary safety outcome of excluding a doubling in the risk of fetal/early infant death as a composite endpoint. In both treatment groups, the rate of fetal/early infant death was low; 1.7% in the 17P and 1.9% in placebo groups (RR = 0.87, 95% CI 0.42–1.81) [10]. Given that the upper bound of the 95% CI was <2.0, a doubling in the risk of fetal/early infant death was excluded.

Table 2 provides the reported rates for miscarriage, stillbirth, and neonatal death from the PROLONG trial. All miscarriages were considered unlikely or definitely not related to study drug by the site investigators. None of the stillbirths were deemed related to study drug by the site investigators. A later evaluation by a Maternal Fetal Medicine (MFM) specialist (co-author BS) of the clinical study report narratives (blinded to treatment) determined that 11 of the 12 stillbirth cases in the 17P group had underlying contributing factors (e.g., infection, abruption, placental infarcts) [16].

Early infant deaths were reported in 0.26% in the 17P vs. 0.18% in placebo groups [10]. None of the early infant deaths (a subset of neonatal deaths) were considered related to study drug by the site investigators.

#### Integrated Meis/PROLONG

The integrated data from both trials for fetal/neonatal death showed a RR of 0.86 (95% CI 0.53–1.41; Table 2), indicating that the risk of this event is unlikely to be different between the two groups.

### Infant follow-up trials

#### Meis trial

A follow-up study of children born to mothers who participated in the Meis trial was conducted to assess any overt differences in child development between the treatment groups. Of 348 eligible surviving children, 278 (80%) were available for evaluation (194 in the 17P group and 84 in the placebo group). The mean age at follow-up was 48 months. A total of 275 children were evaluated using the Ages and Stages Questionnaire (ASQ). There were no significant differences between the groups in overall ASQ or in any of the five domains within the ASQ; there were 53/193 (27.5%) children in the 17P group vs. 23/82 (28.0%) children in the placebo group (*P* = 0.92) scoring positive in at least one ASQ domain. The authors concluded there were no detectable differences in developmental delays, or safety concerns related to overall health or physical examination including genital or reproductive anomalies between children with in-utero exposure to placebo and in-utero exposure to 17P [17].

#### PROLONG

A similar follow-up study (17P-FU-004) of children born to mothers who participated in PROLONG is ongoing. In this study, if a child scores positive on any of the five ASQ domains, they are referred for both a Bayley Scales of Infant and Toddler Development test and a neurological exam. While unblinded PROLONG results are available for the maternal outcomes, and the 28-day follow-up of neonates born to women in the RCT, the companion follow-up study remains blinded to the Sponsor and the contract research organization. As of May 1, 2020, a total of 413 children have been consented to participate by their parent(s)/legal guardian(s). Of these, 288 subjects have reached 22 months of age and, therefore, their parent(s)/legal guardian(s) have been mailed the ASQ version 3. Of the 288 ASQ’s mailed, 232 (80%) questionnaires have been returned to date. Of the 232 received, 49 subjects (21%) have scored positive for developmental delay in at least one of the five ASQ domains and have been referred for Bayley Scales of Infant and Toddler Development and neurological exam. The estimated date for study completion is end of 2020.

## Preclinical safety

In addition to the clinical safety, FDA requested two preclinical safety studies: assessment of reproductive toxicology and investigation of the 17P metabolic pathways to evaluate potential drug-drug interactions. No reproductive or developmental toxicity or impaired fertility was observed in the study with a no-observable-effect level (NOEL) established as 150 mg/kg. 17P demonstrated minimal potential for clinically significant drug interactions of various cytochrome P450 enzymes. Information regarding these preclinical studies is available as a supplement on the JPER website.

## Discussion

17P has been used for more than 60 years in pregnant women. After publication of the Meis trial and subsequent publication of ACOG and the SMFM guidelines [9, 18], use of 17P became the standard of care in the US during the second and third trimester for women with a previous SPTB due to its efficacy and safety profile. Although the PROLONG trial failed to confirm the efficacy of 17P in the population studied, it provided the ability to analyze the safety of 17P by adding data from a large number of women exposed to 17P.

In the Meis trial, there was a numerically higher rate of both miscarriage and stillbirth in the 17P group compared to placebo. While the investigators in the Meis trial did not deem the fetal loss related to 17P, the potential “safety signal” merited closer evaluation in the PROLONG trial. Notably, the PROLONG data demonstrate a lack of association for either miscarriage or stillbirth with 17P use. Furthermore, the point estimate of the RR of the integrated safety for neonatal death is 0.60 (95% CI 0.28–1.28) (Table 2).

Obstetricians have historically considered four primary maternal concerns with progestogens: gestational diabetes, VTE, hypertensive disorders of pregnancy and cholestasis of pregnancy. The integrated data (Table 1) provides reassurance that 17P does not increase the risk of these complications.

Gestational diabetes is particularly clinically relevant to consider, as recent publications including non-randomized studies have raised concern of increased risk of gestational diabetes with 17P [19, 20]. In 2007, Rebarber reported the incidence of gestational diabetes was 12.9% in women who received 17P (*n* = 557) compared with 4.9% in those who did not (*n* = 1524, *P* < 0.001; odds ratio 2.9 [95% CI 2.1–4.1]) [21]. In 2017, Nelson reported the rate of gestational diabetes was 13.4% in women who received 17P compared to 8% in a historical cohort of women from 1988 to 2011 (*P* = 0.001) [22]. In contrast, in 2009, Gyamfi reported rates of gestational diabetes were not different in women receiving 17P vs placebo in the MFMU randomized clinical trial of 17P in twin pregnancies (7.4% 17P vs 7.6% placebo; *P* = 0.94) [23]. The results of PROLONG confirm a lack of association between use of 17P and gestational diabetes, with rates of 3.1% and 3.6% in the 17P and placebo groups, respectively (Table 1).

In assessing the overall safety of 17P, it is also worth noting that a requirement for FDA-approved medications is post-marketing safety surveillance. Nearly 310,000 women have been treated with Makena for the 9-year period from time of drug approval (2011) to February 2020 [24]. The post-marketing safety data remain consistent with the data obtained from the Meis and PROLONG trials; no new safety risks or trends were observed with Makena. While reporting of adverse events by patients and healthcare providers is voluntary, the real-world utilization and lack of safety signals raised via post-marketing surveillance is nevertheless reassuring.

The integrated safety data demonstrates a favorable safety profile of 17P, which was comparable to placebo.

## Supplementary information

Supplement 17P Preclinical Data
