# Supplementary material for: Safety review of hydroxyprogesterone caproate in women with a history of spontaneous preterm birth
Source: J Perinatol. 2020 Oct 14;41(4):718–25. doi: 10.1038/s41372-020-00849-y (PMC8049867; doi:10.1038/s41372-020-00849-y)
Supplement: Supplementary file 1 — Supplement 17P Preclinical Data [file 41372_2020_849_MOESM1_ESM.doc]

**Preclinical Safety**

There were two preclinical safety studies specifically requested by the FDA; one to assess the reproductive toxicology of 17P effects and one to investigate 17P metabolic pathways that may result in drug-drug interactions.

The basis for the first study arose from the numerical, but not statistical, increase in the rate of miscarriages and stillbirths after 17P, as discussed in the Meis trial (1). Prior non-clinical animal studies (2, 3) in mice, rats rabbits, guinea pigs, horses and non-human primates demonstrated developmental or reproductive toxicity in only one species, the rhesus monkey (3). However, another sub-human primate, the cynomolgus monkey, was unaffected when given the same dosage in the same gestation interval (3). Therefore, a multi-generational reproductive toxicology study was designed to address concerns on the possible long-term effects of in utero exposure of 17P to offspring, aligning to the stages of pregnancy when 17P would be administered in humans (4).

The basis for the second study was to characterize previously unexamined effects of 17P metabolism.

**Reproductive Rat Toxicology Study**.

In this GLP-compliant study, 17P was administered intramuscularly to Charles River Laboratory CD strain rats (>400 animals) during three phases of embryo-fetal development: the period of ovarian development (N = 116), following embryo implantation (N = 118), and at a time corresponding to gonadal formation in humans (N = 121) (4). Dose levels up to thirty times the human therapeutic doses, based on a per kilogram basis, were used including 0 (placebo), 5, 25, and 150 mg/kg. When scaled, the 150 mg/kg dose in rats represented approximately five times the human dose on a per meter squared basis.

Data showed that 17P had no adverse effects on the original pregnant dams (F0), their developing offspring (F1), or ability of the F1 to produce viable, normal second (F2) generation offspring. In short, no reproductive or developmental toxicity or impaired fertility was observed in the study with a no-observable-effect level (NOEL) established as 150 mg/kg

**Drug-Drug Interaction Studies**

Two studies evaluating potential drug–drug interactions involving the cytochrome P450 (CYP) enzyme system were conducted by the Sponsor. The first study (Study report 304-1177-02-body, unpublished data) was performed prior to approval of 17P (Makena) to characterize previously unexamined effects of 17P metabolism while the second study (Study-report b11a06, unpublished data) was performed post-approval to better characterize the initial findings on whether 17P induced or altered the metabolic activities of CYP1A2, CYP2A6 and CYP2B6.

The initial human hepatic microsome study determined the potential of 17P to *inhibit* the activities of CYP isoforms (CYP1A2, CYP2A6, CYP2B6, CYP2C8, CYP2C9, CYP2C19, CYP2D6, CYP2E1, and CYP3A4) using ketoconazole, a selective inhibitor, as a positive control. Data showed that the activities of CYP1A2, CYP2A6 CYP2B6, CYP2D6 and CYP2E1 were not inhibited at the tested 17P concentrations (0, 0.02, 0.06, 0.2, 2 and 20 μM). However, 17P stimulated the metabolic rate of CYP1A2, CYP2A6, and CYP2B6 by approximately 80%, 150%, and 80%, respectively. For the other isoforms, 17P did not inhibit activities of CYP2C8, CYP2C9, and CYP2C19 at concentrations up to 2 μM, but inhibited activities at 20 μM concentration. The inhibition ranged from 15.5% to 37.7% and the IC50 value was greater than 20 μM. 17P did not inhibit CYP3A4 activity at concentrations up to 2 μM but inhibited activity at the 20 μM concentration with an IC50 value of approximately 20 μM (Study report 304-1177-02-body, unpublished data).

Since the study suggested induction of three CYP isozymes important in drug metabolism, the product was initially labelled to account for possible drug interactions. Section 7 of the 2011 Makena label stated “the metabolism of drugs metabolized by CYP1A2 (such as theophylline, tizadine, clozapine), CYP2A6 (such as acetaminophen, halothane, nicotine), or CYP2B6 (such as efavirenz, bupropion, methadone) may be increased during treatment with Makena” (5).

The second follow-up study was designed to determine the potential of 17P (0.02, 0.1, 0.5 and 2 μM) to *induce* the activities of CYP1A2, CYP2A6, and CYP2B6 in hepatic microsomes of women to determine the potential of 17P to alter the activities of these isoforms in hepatic microsome of adult women. The data showed that 17P caused no induction or inhibition of any of the CYP activities examined under conditions where the control inducers and inhibitors caused the anticipated increases or decreases in CYP enzyme activities. These investigators concluded that, overall, the findings demonstrated that 17P had minimal or no potential for CYP1A2, CYP2A6, and CYP2B6 related drug-drug interactions at the clinically relevant concentrations (Study-report-b11a06, unpublished data).

The labeling was subsequently amended to “Overall, the findings indicate that hydroxyprogesterone caproate has minimal potential for CYP1A2, CYP2A6, and CYP2B6 related drug-drug interactions at the clinically relevant concentrations. In vitro data indicated that therapeutic concentration of hydroxyprogesterone caproate is not likely to inhibit the activity of CYP2C8, CYP2C9, CYP2C19, CYP2D6, CYP2E1, and CYP3A4” (6).

REFERENCES:

1. Meis PJ, Klebanoff M, Thom E, Dombrowski MP, Sibai B, Moawad AH, et al. Prevention of recurrent preterm delivery by 17 alpha-hydroxyprogesterone caproate. N Engl J Med. 2003;348(24):2379-85.

2. Courtney KD, Valerio DA. Teratology in the Macaca mulatta. Teratology. 1968;1(2):163-72.

3. Hendrickx AG, Korte R, Leuschner F, Neumann BW, Poggel A, Binkerd P, et al. Embryotoxicity of sex steroidal hormones in nonhuman primates: II. Hydroxyprogesterone caproate, estradiol valerate. Teratology. 1987;35(1):129-36.

4. Schardein JL, Birch R, Hesley R, Thorsrud BA. Multigeneration reproductive study of hydroxyprogesterone caproate (HPC) in the rat: laboratory results and clinical significance. Birth Defects Res B Dev Reprod Toxicol. 2012;95(2):160-74.

5. Amag Pharmaceuticals I. MAKENA® (hydroxyprogesterone caproate injection), Prescribing Information. Waltham, MA, USA2011.

6. Amag Pharmaceuticals Inc. Makena (hydroxyprogesterone caproate injection) US Prescribing Information. 2013.
